# DNMT (DNA methyltransferase) inhibitors radiosensitize human cancer cells by suppressing DNA repair activity

**DOI:** 10.1186/1748-717X-7-39

**Published:** 2012-03-20

**Authors:** Hak Jae Kim, Jin Ho Kim, Eui Kyu Chie, Park Da Young, In Ah Kim, Il Han Kim

**Affiliations:** 1 Department of Radiation Oncology, Seoul National University College of Medicine, Seoul, Republic of Korea; 2 Cancer Research Institute, Seoul National University College of Medicine, Seoul, Republic of Korea; 3 Department of Radiation Oncology, Seoul National University Bundang Hospital, Seongnam, Republic of Korea; 4 Institute of Radiation Medicine, Medical Research Center, Seoul National University, Seoul, Republic of Korea

**Keywords:** Cancer, Epigenetics, DNA methylation, DNA methyltransferase inhibitor, Radiosensitization

## Abstract

**Background:**

Histone modifications and DNA methylation are two major factors in epigenetic phenomenon. Unlike the histone deacetylase inhibitors, which are known to exert radiosensitizing effects, there have only been a few studies thus far concerning the role of DNA methyltransferase (DNMT) inhibitors as radiosensitizers. The principal objective of this study was to evaluate the effects of DNMT inhibitors on the radiosensitivity of human cancer cell lines, and to elucidate the mechanisms relevant to that process.

**Methods:**

A549 (lung cancer) and U373MG (glioblastoma) cells were exposed to radiation with or without six DNMT inhibitors (5-azacytidine, 5-aza-2'-deoxycytidine, zebularine, hydralazine, epigallocatechin gallate, and psammaplin A) for 18 hours prior to radiation, after which cell survival was evaluated via clonogenic assays. Cell cycle and apoptosis were analyzed via flow cytometry. Expressions of DNMT1, 3A/3B, and cleaved caspase-3 were detected via Western blotting. Expression of γH2AX, a marker of radiation-induced DNA double-strand break, was examined by immunocytochemistry.

**Results:**

Pretreatment with psammaplin A, 5-aza-2'-deoxycytidine, and zebularine radiosensitized both A549 and U373MG cells. Pretreatment with psammaplin A increased the sub-G1 fraction of A549 cells, as compared to cells exposed to radiation alone. Prolongation of γH2AX expression was observed in the cells treated with DNMT inhibitors prior to radiation as compared with those treated by radiation alone.

**Conclusions:**

Psammaplin A, 5-aza-2'-deoxycytidine, and zebularine induce radiosensitivity in both A549 and U373MG cell lines, and suggest that this effect might be associated with the inhibition of DNA repair.

## Background

Epigenetic alteration is one of the most important gene regulatory mechanisms. Unlike genetic alterations, epigenetic events are not changes in gene function that occur in conjunction with DNA sequence changes. Recently, epigenetic studies have been conducted in many different aspects of biology, and particularly in the cancer field. DNA methylation and histone modifications are two principal factors in epigenetic phenomena. These two mechanisms perform a crucial function in carcinogenesis and tumor progression.

DNA methylation is controlled by DNA methyltransferase (DNMT), an enzyme that catalyzes the transfer of a methyl moiety from S-adenosyl-l-methionine to the 5-postion of cytosines in the CpG dinucleotide [1]. DNMT overexpression has been detected in a variety of malignancies, including lung, prostate, and colorectal tumors [2-4].

Because DNA methylation is a reversible biochemical process, DNMT may be a viable target for the treatment of cancer. Since two cytidine analogues, 5-azacytidine and 5-aza-2'deoxycytidine, have been reported in the 1980s, several DNMT inhibitors are currently under investigation for their possible utility in treating a variety of tumors [5-7].

It has become widely accepted that histone modification and DNA methylation are intricately interrelated in terms of affecting chromatin structure and gene expression [8]. Because these two parameters have long been implicated in the regulation of cellular radioresponse, histone deacetylase (HDAC) inhibitors and DNMT inhibitors might be considered potential targets for radiosensitization. Actually, several studies have reported that HDAC inhibitors such as trichostatin A induce radiosensitization [9-11]. However, relatively little information is currently available concerning the use of DNMT inhibitors in this context [12,13]. This allows us to evaluate the functions of DNMT inhibitors as radiosensitizing agents.

We tried to assess the influence of a variety of DNMT inhibitors on radiosensitivity in two human cancer cell lines of different histologic origins, and to elucidate the mechanisms relevant to those influences.

## Methods

### Cell culture and DNMT inhibitors

In this study, two different cancer cell lines were chosen: A549, a human lung cancer cell line harboring wild-type p53, and U373MG, a human glioblastoma cell line harboring inactive mutant p53. The A549 and U373MG cell lines were purchased from the Korean Cell Line Bank. Cells were cultured at 37°C in water saturated with 5% CO_2_. The cultures were maintained in RPMI media (Welgene, Daegu, Korea), supplemented with 10% fetal bovine serum and 12.5 μg/ml of gentamicin.

5-azacytidine, 5-aza-2'-deoxycytidine, zebularine, hydralazine, epigallocatechin gallate (EGCG), and psammaplin A were obtained from Sigma Chemical Co. (St. Louis, MO, USA), and dissolved as concentrated stock solutions in DMSO, stored at -20°C, and diluted in the respective culture media at the time of use. Control cells were treated with media containing an equal concentration of the drug carrier, DMSO.

### Clonogenic assay

Cells were trypsinized from the exponentially growing monolayer cultures. The appropriate numbers of cells were seeded into T25 flasks, and then incubated for 24 hours prior to treatment. To compare the combined cytotoxic effect of DNMT inhibitors and radiation with that of radiation alone, radiation was administered with 6 MV of x-rays from a linear accelerator (Clinac 2100 C or Clinac 21EX, Varian Medical systems, Palo Alto, CA, USA) with graded doses of x-rays. In combined treatment, DNMT inhibitors (5-azacytidine, 5-aza-2'-deoxycytidine, zebularine, hydralazine, EGCG and psammaplin A) were administered for 18 hours prior to radiation. Our previous studies for epigentics, in which HDAC inhibitors had been used, showed that the greatest degree of radiosensitization was observed when cells are pretreated with epigenetic drugs before irradiation for 18 hours. Based on these results, all subsequent experiments were done with DNMT inhibitors added before radiation for 18 hours [10]. To cancel out additive cytotoxicity of DNMT inhibitor and reveal synergism with radiation, we normalized surviving fractions of irradiated cells by using corresponding plating efficacies of unirradiated cells within respective groups. The cells were incubated for 14 to 21 days to allow for the formation of colonies. The colonies formed were fixed with methanol and stained with 0.5% crystal violet; the number of colonies containing at least 50 cells was determined, and the surviving fraction (SF) was then calculated. Each point on the survival curves represents the mean surviving fraction from at least three dishes.

### Flow cytometric analysis

The effects of DNMT inhibitors on cell cycles were analyzed via flow cytometry. All centrifugation procedures were conducted for 4 min at 1500 rpm. The cells were treated with RPMI or an IC_50_s concentration of DNMT inhibitors for 18 hours. The cells were sham-irradiated or irradiated with 6 Gy of 6 MV x-ray and were collected at 0, 2, 6, 12 and 24 hours after radiation. The cells were harvested at the indicated times and fixed in 2 mL of 80% ethanol for 24 hours for fixation. The fixed cells were then washed twice in PBS, and suspended in PBS on ice for 5 min. After centrifugation, the cell pellets were washed and resuspended in 5 μg/ml propidium iodide (Molecular Probes, Eugene, OR, USA) and 0.1% RNase A (Sigma). At least 1 × 10^4 ^events were counted.

### Western blot for DNMT1, DNMT3A/3B, and cleaved caspase-3

Cell lysates were prepared in cell lysis buffer (iNtRON Biotechnology, Seoul, Korea). The total cellular proteins (50 μg) were separated on SDS-PAGE and transferred to nitrocellulose membranes (Millipore Corp., Bedford, MA, USA). The membranes were blocked with blocking solution in 5% nonfat dry milk (25 mM Tris, pH 7.5; 0.15 M NaCl; 0.05% Tween) for 1 hour and probed with primary rabbit polyclonal IgG antibody at a dilution of 1:1,000 overnight. The antibody for DNMT1 was obtained from Abcam (Cambridge, UK), and DNMT3A, DNMT3B and cleaved caspase-3 antibody were purchased from Cell Signaling Technology (Beverly, MA, USA). The membranes were incubated with blocking solution containing a dilution of HRP-conjugated goat anti-rabbit IgG as a secondary antibody (Santa Cruz, Biotechnology, CA, USA) at 1:2,000 for 2 hours. Western blot protein detection was conducted using the ECL kit (Intron Biotechnology, Seongnam, Korea) according to the manufacturers' recommendations. As a control, monoclonal antibody against actin (Santa Cruz) was utilized.

### Immunocytochemistry

Cells were grown and treated in tissue culture chamber slides (Nalge Nunc International, Naperville, IL, USA). At the specified times, the medium was aspirated and the cells were fixed in 4% paraformaldehyde for 10 minutes at room temperature. The paraformaldehyde was aspirated, and the cells were treated for 15 min with a 0.2% NP40/PBS solution. The cells were then washed twice in PBS, and anti-γH2AX antibody (Cell Signaling Technology) was added at a dilution of 1:200 in 1% bovine serum albumin and incubated overnight at 4°C. The cells were again washed twice in PBS prior to 1 hour of incubation in the dark with an FITC-labeled secondary antibody (Invitrogen, Camarillo, CA, USA) at a dilution of 1:50 in 1% bovine serum albumin. The secondary antibody solution was then aspirated and the cells were washed twice in PBS, followed by 30 minutes of incubation in the dark with 4',6diamindino-2-phenylindole (1 μg/mL) in PBS and two subsequent washings. The coverslips were then mounted with antifade solution (Vector Laboratories, Burlingame, CA, USA). Slides were examined with a Leica DMRXA fluorescent microscope (Leica, Wetzlar, Germany). The images were captured using a Photometrics Sensys CCD camera (Leica) and imported into the IP Labs image analysis software package (Leica). For each treatment condition, the numbers of γH2AX foci were counted in 50 cells. Cells were classified positive (*i.e.*, containing radiation-induced γH2AX foci) when more than five foci were detected [14].

### Statistics

Kaleidagraph version 3.51 (Synergy Software, Reading, PA, USA) was utilized to fit the survival data of irradiated cells into a linear quadratic (LQ) model and to estimate the α and β values. The LQ model was defined as follows:

$$\text{SF}={\text{e}}^{\text{ - }\left(\text{α}\text{d }+\text{β}\text{d2}\right)}$$

Differences in mean values between groups were compared using Student t-test. Probability values of *p *< 0.05 were regarded as statistically significant.

## Results

### Cytotoxic effect and determination of IC_50_s of DNMT inhibitors

Increasing concentrations of six DNMT inhibitors reduced the viability of two cell lines. The 50% inhibitory concentrations (IC_50_s) were determined after an 18-hour exposure to six DNMT inhibitors, and those concentration were used in subsequent experiments. The IC_50_s of DNMT inhibitors are shown in Table 1. IC_50 _value of zebularine in A549 cells could not be obtained because no significant inhibition of cell growth was observed at any dose. Thus, IC_50 _value in U373MG, 800 uM, was used in subsequent experiments in A549 cells.

**Table 1:** IC_50 _values for six DNMT inhibitors

Drugs	Cell lines	
	A549	U373MG
5-azacytine	3 uM	3 uM
5-aza-2'-deoxycytidine	300 nM	100 nM
Zebularine	800 uM	ND*
Hydralazine	2 uM	20 uM
Epigallocatechin gallate	15 uM	5 uM
Psammaplin A	5 ug/ml	5 ug/ml

*ND; not determined, IC_50 _value in U373MG, 800 uM, was used in subsequent experiments in A549 cells

### Effects of DNMT inhibitors on radiosensitivity of A549 and U373MG cell lines

**Figure 1:**
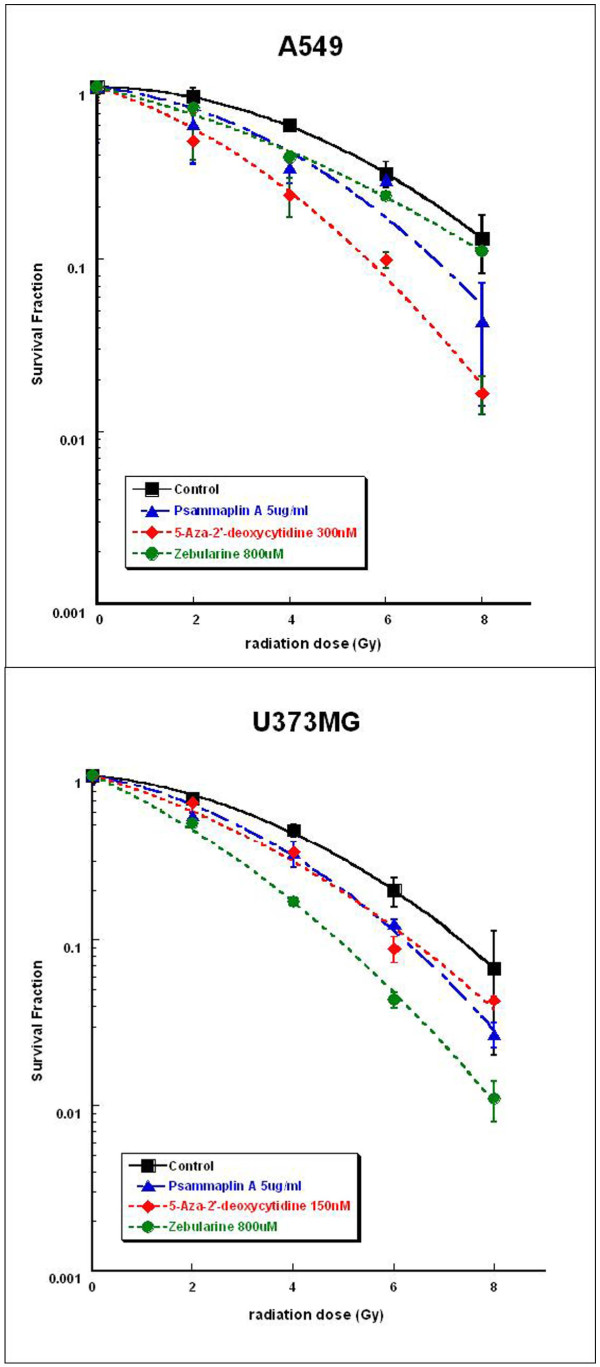
**The effects of DNMT inhibitors on tumor cell radiosensitivity**. Survival curves of (A) A549 cells and (B) U373MG cells treated with the respective DNMT inhibitors prior to radiation and radiation were compared with those of radiation alone. Points, mean for three independent experiments; bars, SE.

Survival curves of A549 and U373MG cells treated with DNMT inhibitors and radiation were compared with those of cells treated with radiation alone. Among six DNMT inhibitors, psammaplin A, 5-aza-2'-deoxycytidine, and zebularine pretreatment significantly enhanced radiation cell killing in both A549 and U373MG cells lines (Figure 1A and 1B). Using the dose required to generate a SF of 0.5 as a reference, the dose enhancement ratios (DER) were estimated. The DERs of psammaplin A, 5-aza-2'-deoxycytidine, and zebularine for the A549 cell line were 1.29, 1.96, and 1.69, respectively and for U373MG were 1.29, 1.55, and 2.13, respectively.

### Effects of DNMT inhibitors on DNMT expression

**Figure 2:**
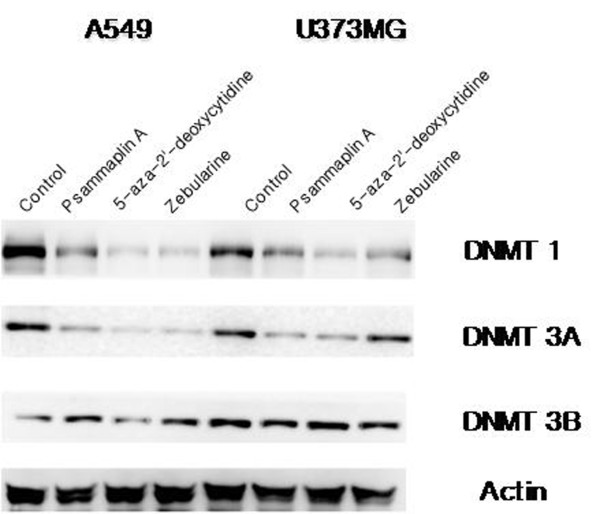
**Methylation status was determined after exposure to DNMT inhibitors using Western blot analysis of DNMT1, 3A/3B**. The cells were treated with DNMT inhibitors for 18 hours. A drastic depletion of DNMT1 and DNMT3A by psammaplin A, 5-aza-2'deoxycytidine, and zebularine in both A549 and U373MG cell lines was observed. However, there was no depletion of DNMT3B by the three DNMT inhibitors in either of the cell lines. Each blot is representative of two independent experiments, with actin used as a loading control.

DNMT1, DNMT3A, and DNMT3B are the three main functional methyltransferases responsible for the establishment and maintenance of DNA methylation patterns in mammals. The effects of DNMT inhibitors on the levels of DNMT expression were analyzed via Western blotting using specific antibodies against DNMT1, DNMT3A, and DNMT3B. Western blot analysis revealed a drastic depletion of DNMT1 and DNMT3A by psammaplin A, 5-aza-2'-deoxycytidine, and zebularine in both A549 and U373MG cell lines. However, no depletion of DNMT3B by the three DNMT inhibitors was observed in either of the cell lines (Figure 2). These results indicate that DNMT inhibitors induce selective demethylation in each of the evaluated tumor cell lines.

### Mechanisms of radiosensitization

**Figure 3:**
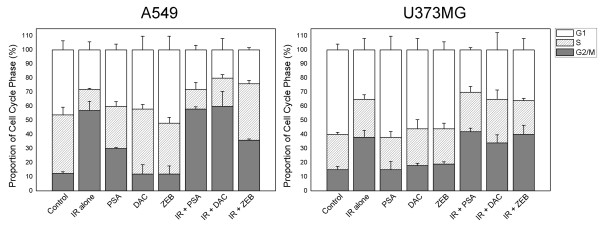
**Influence of DNMT inhibitors on cell cycle phase distributions of A549 and U373MG cells**. Cell cycle phase was measured using flow cytometry at 12 hours after 6 Gy of radiation. Columns, proportion of cell cycle phase; bars, SE.

**Figure 4:**
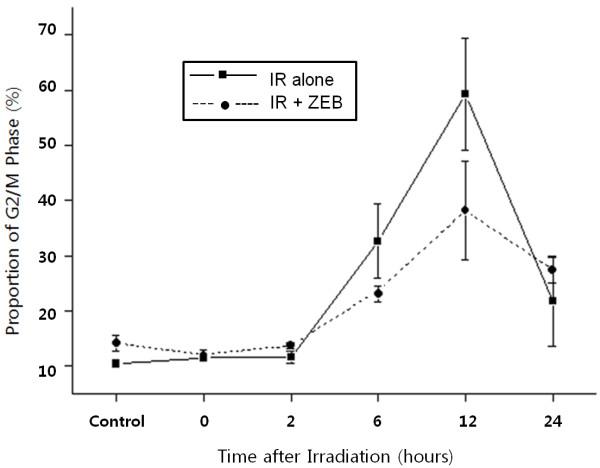
**The effect of zebularine on radiation-induced G2/M arrest in A549 cells**. A549 cells were treated with 800 uM zebularine for 18 hours and then irradiated with 6 Gy. A549 cells were accumulated in G2/M phase after radiation. This radiation-induced G2/M arrest was abrogated by zebularine pretreatment at 6-12 hours, but this abrogation disappeared at 24 hours.

Cell cycle and apoptosis were evaluated by flow cytometry. Both cell lines evidenced a G2/M delay after radiation treatment alone. Although the effects of combining DNMT inhibitors and radiation vary between cell lines, we noted no significant differences in cell cycle phase distribution patterns between cells treated with radiation alone and those treated with a combination of radiation combined with DNMT inhibitors (Figure 3). However, in the A549 cells, radiation-induced G2/M arrest was abrogated by zebularine pretreatment at 6-12 hours, but this abrogation disappeared at 24 hours (Figure 3 and 4).

**Figure 5:**
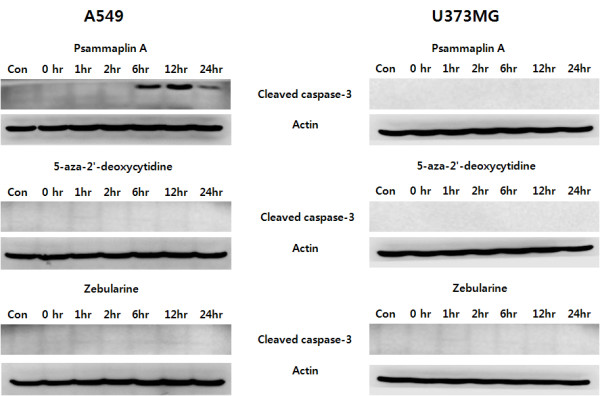
**Western blot analysis of cleaved caspase-3**. The cells were treated with a combination of three different DNMT inhibitors and 6 Gy of radiation. Increased cleaved caspase-3 protein level was observed in cells treated with a combination of psammaplin A and 6 Gy of radiation in the A549 cells.

Psammaplin A pretreatment increased the sub-G1 fraction of A549 cells, as compared to cells exposed to radiation alone (Table 2). Western blotting also revealed an increased expression of cleaved caspase-3 in psammaplin A pretreated A549 cells (Figure 5). However, psammaplin A exerted no effect on the apoptotic induction of U373MG cell lines. Pretreatment with other DNMT inhibitors did not influence the expression of cleaved caspase-3 in either of the cell lines.

**Table 2:** Apoptotic rates measured by the sub-G1 portion of flow cytometry in A549 and U373MG cells

		Control/drug	0 h	2 h	6 h	12 h	24 h
A549	IR	1.11 ± 0.93	1.55 ± 0.82	3.01 ± 1.93	1.72 ± 0.83	1.20 ± 0.99	1.03 ± 0.33
	PSA + IR	2.61 ± 1.05	1.53 ± 0.73	3.29 ± 1.71	8.02 ± 2.49	9.02 ± 3.20	7.72 ± 2.16
	IR	0.53 ± 0.10	1.23 ± 1.07	0.98 ± 0.37	0.70 ± 0.06	1.13 ± 0.88	0.89 ± 0.12
	DAC + IR	0.43 ± 0.10	1.20 ± 0.70	0.54 ± 0.02	0.57 ± 0.09	0.53 ± 0.07	1.05 ± 0.14
	IR	0.59 ± 0.16	0.94 ± 0.60	0.61 ± 0.15	0.86 ± 0.33	1.77 ± 0.66	1.66 ± 0.14
	ZEB + IR	0.84 ± 0.32	1.00 ± 0.61	0.97 ± 0.49	1.65 ± 0.52	1.62 ± 0.14	2.13 ± 0.05
U373MG	IR	0.95 ± 0.10	0.61 ± 0.23	0.80 ± 0.18	0.71 ± 0.09	1.39 ± 0.20	1.68 ± 0.55
	PSA + IR	0.71 ± 0.19	0.67 ± 0.13	0.55 ± 0.06	0.63 ± 0.05	0.89 ± 0.16	1.57 ± 0.61
	IR	0.94 ± 0.05	0.64 ± 0.13	0.61 ± 0.05	0.64 ± 0.05	0.99 ± 0.20	1.68 ± 0.65
	DAC + IR	0.77 ± 0.04	0.83 ± 0.01	0.58 ± 0.05	0.81 ± 0.08	0.93 ± 0.12	1.45 ± 0.37
	IR	1.58 ± 0.25	1.39 ± 0.34	1.35 ± 0.01	1.12 ± 0.21	1.78 ± 0.34	2.33 ± 0.35
	ZEB + IR	0.95 ± 0.19	1.90 ± 1.21	2.14 ± 1.48	1.06 ± 0.01	1.28 ± 0.15	4.59 ± 1.23

*Abbreviations: *IR = irradiation, PSA = psammplin A, DAC = 5-aza-2'-deoxycytidine, ZEB = zebularine

**Figure 6:**
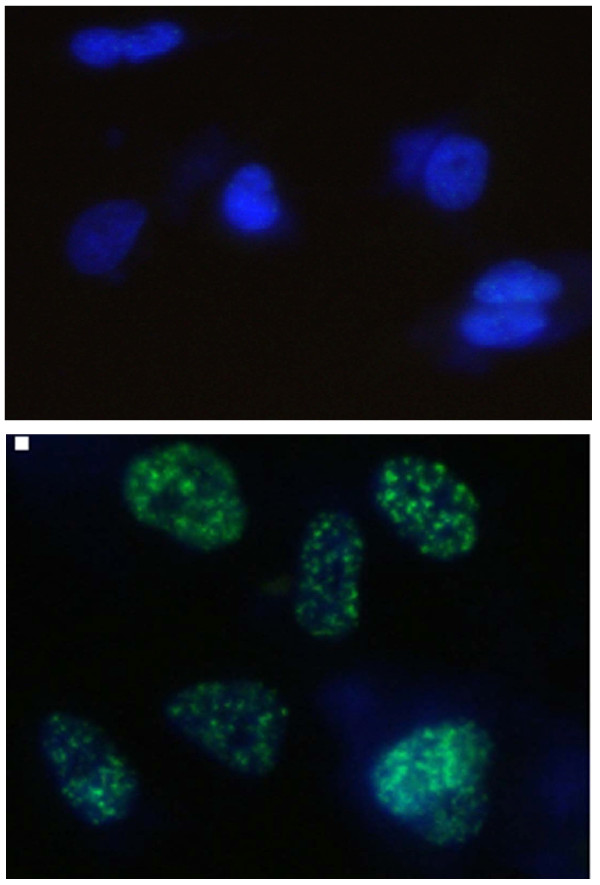
**Radiation-induced γH2AX foci**. Representative micrographs obtained from (A) control cells and (B) cells that had received 6 Gy of radiation 1 hour earlier. (A) top panel, (B) bottom panel.

**Figure 7:**
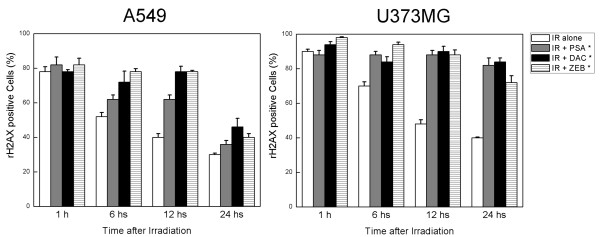
**Influence of DNMT inhibitors on radiation-induced γH2AX foci**. A549 and U373MG cells growing in chamber slides were exposed to DNMT inhibitors for 18 hours, irradiated, and fixed at specific times for immunocytochemical analyses of nuclear γH2AX foci. Open columns, data from cells receiving radiation alone; grey columns, data from cells that were exposed to psammaplin A and radiation; filled columns, data from cells that were exposed to 5-aza-2'-deoxycytidine and radiation; hatched columns, data from cells that were exposed to zebularine and radiation. Cells with more than five foci per nucleus were classified as positive for radiation-induced γH2AX. Foci were evaluated in 50 nuclei. Bars, SE. *p < 0.01 as determined by a logistic regression compared with radiation alone (6 Gy) group.

γH2AX has been identified as a marker of DNA double-strand break (DSB) [15]. Immunocytochemical analysis using the anti-γH2AX antibodies was conducted in order to determine the effects of DNMT inhibitors on DNA repair. As shown in the representative micrographs in Figure 6, γH2AX foci could be clearly distinguished after the 6 Gy of radiation. The γH2AX expression of cells treated with radiation alone was compared with those treated with a combination of DNMT inhibitors and radiation. Although γH2AX foci expression was shown to be reduced in cells treated with radiation alone over time, γH2AX foci levels in the cells exposed to DNMT inhibitors prior to radiation remained constant over a 12-hour time course in A549 cells and over a 24-hour time course in U373MG cells (Figure 7). These results implicate an inhibition of the DNA damage repair process as the possible mechanism underlying the effects of DNMT inhibitors on radiosensitization.

## Discussion

Recently, histone modifications and DNA methylation, both of which are prominent epigenetic mechanisms, have been evaluated with a view toward enhancing the radiosensitivity of tumor cells via the regulation of chromatin structure modifications and the expression of genes involved in cell cycle checkpoints, apoptosis, and DNA repair. A number of investigators have previously reported that several HDAC inhibitors exert direct cytotoxic effects, and can sensitize tumor cells to radiotherapy [14,16-19]. Kim *et al. *also demonstrated that trichostatin A, which is the most potent HDAC inhibitor identified thus far, enhanced radiosensitivity in a variety of human cancer cell lines [11,20]. Unlike the HDAC inhibitors, however, little information is currently available regarding the effects of DNMT inhibitors on radiosensitization [12,13].

There are two classes of DNMT inhibitors: the nucleoside analogues and the non-nucleoside analogues. Several compounds are currently being evaluated in preclinical and clinical trials for the treatment of solid and hematological malignancies. Contrary to their effect in hematologic malignancies, the clinical effects of these compounds against solid tumors have yet to be evaluated [21]. Therefore, the use of epigenetic therapies in combination with other regimens, such as conventional chemotherapeutics or radiotherapy, should be considered. As DNA methylation is intricately interrelated with chromatin structure and gene expression, parameters long thought to be involved in the regulation of cellular radioresponse, it is critically important to evaluate the effects of DNMT inhibitors on radiosensitization. In order to assess this possibility, we employed a total of six DNMT inhibitors. Among them, 5-aza2'-deoxycytidine, zebularine, and psammaplin A were shown to exert a radiosensitizing effect in both A549 and U373MG cells. Although some differences in the degrees of radiosensitization were detected according to the different DNMT inhibitors and cell lines used, these results demonstrate that some DNMT inhibitors may prove to be useful radiation sensitizers in human cancer cells.

5-aza-2'-deoxycytidine, along with 5-azacytidine, is the first DNMT inhibitor reported, and evidences clinical efficacy in cases of myelodysplastic syndrome and acute myelogenous leukemia. Several previous studies have demonstrated that 5-aza-2'-deoxycytidine exerts a radiosensitizing effect, with or without HDAC inhibitors, in a variety of cancer cell lines [13,22,23]. Recently, the radiosensitization effect of 5-azacytidine was also referenced by Hofstetter *et al.*, who demonstrated that 5-azacytidine-induced genomic hypomethylation induces enhanced radiation sensitivity in colorectal carcinoma [24]. These results are logical consequences, considering that 5-azacytidine has a molecular structure and exerts clinical effects similar to those of 5-aza-2'-deoxycytidine. However, in this study, 5-azacytidine did not sensitize either of the tested cancer cell types to radiotherapy. 5-azacytidine may exert differing effects on radiosensitivity according to cell type, and the IC_50 _of 5-azacytidine varies among different cell lines. Although this may explain, at least in part, the different results detected in our study, we did not successfully generate a definitive explanation for the absence of any detectable radiosensitizing effect of 5-azacytidine. Because very few studies have addressed the role of 5-azacytidine as a radiosensitizer, further studies will be required to resolve this issue.

Zebularine, another 5-azacytidine derivative, was also shown in this study to induce radiosensitivity. Zebularine has been demonstrated to have anticancer properties, which can cause re-expression of the epigenetically silenced tumor suppressor p16 in solid malignancies. However, its clinical development is limited by its higher dosage and poor bioavailability, as observed previously in rats, mice, and monkeys [6,25,26]. Owing to the relatively high toxicity of these nucleoside analogue compounds, non-nucleoside DNMT inhibitors are currently being investigated for their possible use as anticancer agents.

The natural marine product, psammaplin A, was initially isolated from the *Psammaplysilla *sponge in 1987. Since that time, psammaplin A has been shown to exert potent cytotoxicity against several cancer cell lines, via the selective induction of genes associated with cell cycle arrest and apoptosis [27,28]. Because psammaplin A has already been shown to exhibit potent histone deacetylase inhibitory activity, this drug is a promising radiosensitizing agent [28]. This is the first study in which the radiosensitizing effects of psammaplin A have been confirmed. As psammaplin A has not yet been the subject of clinical trials, further clinical research should be conducted to determine its efficacy when administered in combination with radiotherapy.

Three different DNMTs exist, all of which are viable targets for DNMT inhibitors. DNMT1, the most abundant of the three, is responsible for methylation during DNA replication (maintenance methyltransferase). Other known methyltransferases include DNMT3A and DNMT3B, which exhibit identical preferences for hemi-methylated and non-methylated DNA, and have thus been classified as *de novo *methyltransferases. However, it has been demonstrated that DNMT1 and DNMT3A/3B do not appear to be completely distinct in their activities [29]. This supposition is based on two lines of evidence: first, despite being DNMT1-deficient, a colon adenocarcinoma cell line was shown to be capable of retaining 80% of its methylation level while replicating, which implies that DNMT3 may perform a function in the maintenance of methylation [30]; secondly, forced DNMT1 overexpression in cancer cell lines does, indeed, induce *de novo *methylation [31]. Gene silencing of DNMT, which functions as an oncogene, has been proposed as a good cancer treatment strategy [32]. In this study, three different DNMT inhibitors, all of which demonstrated radiosensitizing effects, depleted DNMT1 and DNMT3A, but no such DNMT3B depletion was noted in the A549 and U373MG cell lines. Depletion of DNMT, along with several members of the structural maintenance of chromatin proteins (SMCs), SMC-associated protein, and heterochromatin proteins, was reported to correlate with chromatin decondensation [33]. Considering that the effect of chromatin compaction on protection of DNA against radiation-induced DSBs has been relatively well-established [34,35], the relaxation of chromatin structure via the downregulation of DNMT with DNMT inhibitors may be a possible mechanism for radiosensitization.

Selective inhibition of DNMT isoforms shown in this study may be attributable, in part, to the doses of DNMT inhibitors. Beaulieu *et al. *previously reported that Western blot analysis with anti-DNMT1, DNMT3A, or DNMT3B antibodies treated with increasing doses of DNMT inhibitors effected a selective dose-dependent inhibition of the target isoform of DNMT [36]. However, as only the IC_50 _values of the DNMT inhibitors were employed in this study, we were unable to ascertain whether this selective inhibition of DNMT isoforms was a function of the concentration of the drug.

In addition to the physical modification of chromatin structure, cellular processes that can affect intrinsic radiosensitivity were evaluated in this study. Cell cycle, apoptosis, and DNA repair have all been shown to influence radiosensitivity.

The proportion of cells in the G2/M phase detected in this study was not significantly altered by pretreatment with DNMT inhibitors, and no abrogation of radiation-induced G2/M arrest was noted except in the zebularine-treated A549 cell line. Radiation-induced G2/M arrest was abrogated as the result of zebularine pretreatment at 6-12 hours, but this effect disappeared at 24 hours in the A549 cell line. In another study, zebularine was not found to result in the abrogation of radiation-induced G2/M arrest in a U251 glioblastoma cell line [12]. Thus, although there appears to be some relationship between cell lines and radiation-induced G2/M arrest, it is less likely that the radiosensitization induced by the DNMT inhibitors employed in this study is the result of a general inhibition of G2 checkpoint activation.

Apoptosis has previously been regarded as a potential mechanism for radiosensitization. Different results have been reported regarding the role of apoptosis as a radiosensitizing mechanism induced by DNMT inhibitors. Dote *et al. *previously reported that a combination of radiation and zebularine did not significantly increase the sub-G1 population (apoptotic cells) [12]. On the other hand, Qui *et al. *demonstrated that 5-aza-2'-deoxycytidine induces radiosensitization in certain gastric cancer cell lines via induced increases in the apoptotic rate, as evidenced by enhanced expression of the p53, RASSF1, and DAPK gene families [23]. In this study, the addition of 5-aza-2'-deoxycytidine or zebularine to radiation did not increase the apoptosis rate in either of the cell lines. However, exposure to psammaplin A in the A549 cell line induced a significant increase in apoptotic death. Psammaplin A has been reported to exert cytotoxic effects on cancer cells via the selective induction of apoptosis-related genes [27]. This effect of psammalin A may result in an increase in radiation-induced apoptosis. However, in this study, radiation-induced apoptosis was noted only in the A549 cell line, and not in the U373MG cell line. This finding may be attributable, at least in part, to p53 expression status. The U373MG cell line, which contains mutated p53, might be comparatively resistant to radiation-induced apoptosis, considering that apoptosis is mediated by the p53 protein.

DNA repair is another process involved in the determination of cellular radiosensitivity. The activation of DNA repair of cancer cells after sublethal DNA damage induced by radiation might be one of the most important factors in resistance. The expression of γH2AX has been recently identified as a sensitive indicator of radiation-induced DSBs [37]. In this study, γH2AX expression in cells treated with a combination of DNMT inhibitors (5-aza-2'deoxycytidine and psammaplin A, as well as zebularine) was found to be similar to the results achieved with radiation treatment at 1 hour after initial treatment, but was significantly greater over time. This finding is generally consistent with the results obtained in other previous studies, in which zebularine and 5-aza-2'-deoxycytidine were employed as radiosensitizing agents [12,22]. Dote *et al. *evaluated the expression of γH2AX foci after exposure to 2 Gy with and without zebularine pretreatment. Whereas zebularine had no effect on radiation-induced γH2AX foci at 1 hour, the number of γH2AX per cell was significantly greater in the zebularine-treated cells at 24 hours after irradiation, which suggested the possible presence of unrepaired DNA damage [12]. DNA methylation is intimately associated with histone deacetylases in terms of the epigenetic regulation of gene expression. Actually, HDAC inhibitors such as LBH589 and MS-275 have been shown to enhance radiosensitivity through similar mechanisms as those of the DNMT inhibitors. These HDAC inhibitors prolonged γH2AX expression, suggesting an inhibition of DNA repair [14,38]. A previous preclinical study demonstrated that an HDAC inhibitor downmodulated the expression of DNA-PK and Rad51, which participated in the recovery of DSB, thereby abrogating key cellular pathways involved in DNA DSB repair [17]. These results indicate that the impairment of DNA DSB repair may be one of the most crucial mechanisms underlying enhanced radiation responses in epigenetic phenomena. Based on this assumption, further investigations are warranted to determine whether or not alterations in the methylation patterns of a specific gene or set of genes involved in DNA repair might be modulated by DNMT inhibitors, and that these changes might contribute to the observed enhancements of radiosensitivity.

Several remain to be determined in future studies. First, a unified treatment schedule - specifically, the administration of DNMT inhibitors 18 hours prior to radiation - was employed in this study, and thus the optimal treatment schedule of DNMT inhibitors and radiation remains to be established. In other studies, DNMT inhibitors were administered for different treatment durations, i.e. 2 ~ 48 hours prior to radiation, considering several factors such as drug half-lives and the expression of radiosensitivity-related genes [22,23]. Further investigations into the optimal treatment schedule of DNMT inhibitors and radiation for clinical applications will be necessary in the future.

Second, another important issue will involve assessments of the synergistic effects of DNMT inhibitors and HDAC inhibitors on radiosensitivity. Although some previous studies have reported that the combined gene silencing reversal effect was superior to that of treatment with a single agent [32,39], only a few studies have thus far evaluated the influence of this combined effect on increased radiosensitivity. Third, the mechanisms underlying DNMT inhibitor-induced radiosensitzations need further investigation. We speculate that suppression of DNMTs by the DNMT inhibitors was associated with enhanced radiosensitivity through the change in DNA structure. However, the relationship between DNA methylation and cellular radiosensitivity is to be elucidated in the future study.

## Conclusions

Taken together, our study indicate that psammaplin A, 5-aza-2'-deoxycytidine, and zebularine have the potential to increase radiosensitivity in lung cancer A549 and glioblastoma U373MG cells, most probably by modulating the impairment of the DNA repair process. Further investigations will be required to identify other additional mechanisms associated with radiosensitivity, and to confirm the synergistic effects on radiosensitivity with other epigenetic drugs, such as the HDAC inhibitors. Also, future studies should be conducted to determine definitively whether the combination of DNMT inhibitors and radiation has real potential as a clinical strategy for the treatment of cancer.

## Competing interests

The authors declare that they have no competing interests.

## Authors' contributions

HJK, IHK, Idea and study design, JHK, IAK Analysis and development of methods. HJK, Manuscript writing. DYP, Technique work. All authors, Review and final approval.
